# Early human development and stem cell-based human embryo models

**DOI:** 10.1016/j.stem.2024.09.002

**Published:** 2024-10-03

**Authors:** Marta N. Shahbazi, Vincent Pasque

**Affiliations:** 1 MRC Laboratory of Molecular Biology, Cambridge CB2 0QH, UK; 2 Department of Development and Regeneration, KU Leuven, 3000 Leuven, Belgium; 3 Leuven Stem Cell Institute & Leuven Institute for Single-Cell Omics (LISCO), Leuven, Belgium

## Abstract

The use of stem cells to model the early human embryo promises to transform our understanding of developmental biology and human reproduction. In this review, we present our current knowledge of the first 2 weeks of human embryo development. We first focus on the distinct cell lineages of the embryo and the derivation of stem cell lines. We then discuss the intercellular crosstalk that guides early embryo development and how this crosstalk is recapitulated *in vitro* to generate stem cell-based embryo models. We highlight advances in this fast-developing field, discuss current limitations, and provide a vision for the future.

## Introduction

Our understanding of early human embryo development remains limited, mainly due to the restricted availability of human embryos for research and the lack of complete conservation of developmental mechanisms across species. Further insights are needed to advance fundamental biology, improve assisted reproductive technology (ART), prevent pregnancy loss, and identify the onset of congenital defects and developmental origins of adult diseases.

Recent advances have come from the establishment of stem cell lines representative of early human embryo lineages.^1,2^ Human pluripotent stem cells (PSCs) possess a high degree of cellular plasticity, which has enabled strategies to guide PSCs into multiple embryonic and extra-embryonic cell types.^3–5^ Remarkable progress has been made in modeling the human and non-human primate embryo by utilizing the self-organizing capabilities of human stem cells.^6–13^ These models partially recapitulate specific stages of early human embryo development *in vitro* and have started to inform us on the mechanisms of cell fate specification and intercellular communication. However, the models do not fully recapitulate the embryo and do not replace, but instead complement, research on human embryos. Collectively, human stem cell-based embryo models promise to yield considerable insights into the key mechanisms of human embryogenesis.

In this review, we focus on the mechanisms of cell fate specification during the first 2 weeks of human embryo development. We discuss the signaling pathways and transcription factors that control cell identity and explore how these factors are modulated by cell-cell communication in the embryo. We present the stem cell types and stem cell-based embryo models, their advantages and limitations, and highlight efforts to overcome bottlenecks.

## Pre-Implantation Development

### From zygote to blastocyst

**Figure 1:**
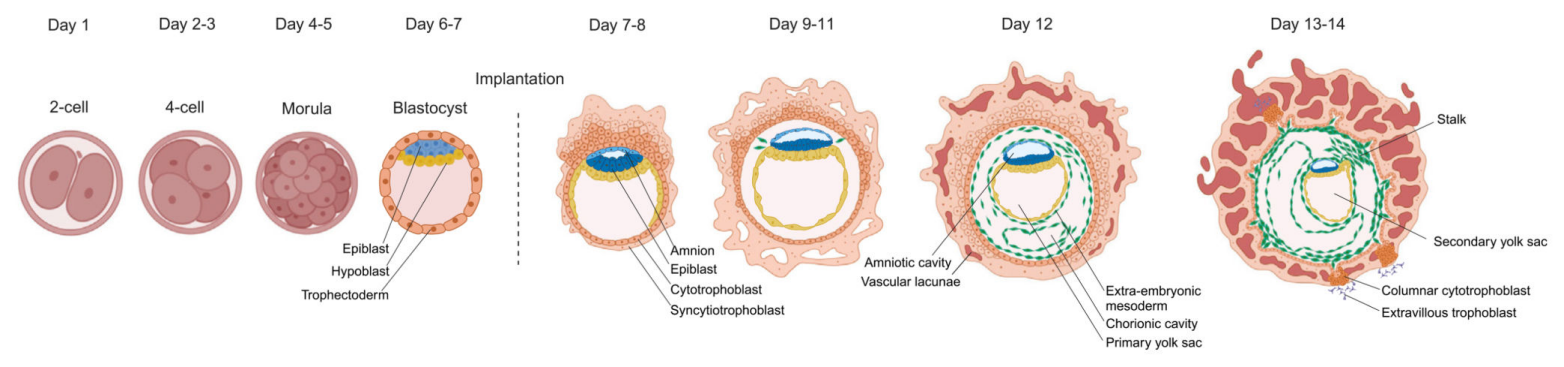
Human embryo development timeline from day 0 to day 14 Overview of the cell types and morphology of the human embryo during the first 2 weeks of human embryo development.

Human embryonic development starts with the formation of the zygote, which undergoes cleavage divisions to form the embryo and extra-embryonic tissues. When human embryos have on average ten cells, compaction leads to the formation of the morula^14^ (Box 1). Subsequently, at around the 20-cell stage, fluid is pumped between the cells that form the embryo, initiating the formation of a cavity. At this point, the embryo reaches the early blastocyst stage (Figure 1). The blastocyst undergoes several cycles of collapse and expansion until it reaches the late blastocyst stage, when the embryo hatches,^15^ comprising about 200 cells,^16^ and is ready for implantation.

Until the eight-cell stage, development is mainly driven by maternally inherited factors. However, activation of embryonic transcription between the four and eight-cell stage marks the start of a gene regulatory program and morphogenetic events at the basis of cell fate specification and self-organization.^19^ The three founding lineages, trophectoderm, epiblast, and hypoblast (Box 1), are established during the first week of development. A first cell fate decision in outer cells at the morula stage generates polarized epithelial cells of the trophectoderm, the tissue of origin of the placenta, while inner cell mass (ICM) cells remain apolar.^20–22^ In the late blastocyst, cells of the ICM undergo a second lineage decision that segregates epiblast and hypoblast cells.^23,24^ Epiblast cells are the pluripotent cells that produce all fetal tissues, whereas hypoblast cells, also called primitive endoderm cells, give rise to the extra-embryonic endoderm of the yolk sac.^25,26^

#### First cell fate decision: Trophectoderm versus ICM

The first cell fate decision segregates the trophectoderm from the ICM through the interplay between morphogenetic events, signaling, and gene regulatory programs. At approximately the ten-cell stage, apical polarity determinants, such as atypical protein kinase C (aPKC), become localized to cell contact-free domains,^20,21^ while the cell-cell adhesion protein E-cadherin becomes basolaterally enriched.^27^ Acquisition of apicobasal polarity in outer cells is coupled to HIPPO signaling, which triggers initiation of the first cell fate decision. Mechanistically, HIPPO signaling, through its downstream kinases LATS1/2 is inhibited in outer polarized cells.^28^ As a result, transcription factor YAP1 localizes to the nucleus of outer cells and, together with TEAD4,^20^ activates the transcription factor GATA3, thereby initiating the trophectoderm program. Inner cells show cytoplasmic YAP1, while GATA3 and the trophectoderm program are repressed. Consistent with this model, blocking polarity or depleting GATA3 affects trophectoderm specification.^20,21^ Another study has found that TEAD4 regulates trophectoderm differentiation upstream of CDX2 in a GATA3-independent manner.^29^ TEAD1 has also been implicated in trophectoderm specification.^22^ The stepwise mechanism of human trophectoderm specification remains incompletely understood.

The existence of an intermediate ICM stage that precedes the segregation of epiblast and hypoblast cells has been confirmed by several studies.^22,27,30,31^ ICM cells are characterized by expression of LAMA4, IFI16 (though also expressed in epiblast), and PDGFRA (expressed in ICM, then restricted to the hypoblast), and lack of aquaporin-3 (AQP3) expression.^23,27,32^ A pioneering study used CRISPR genome editing in human embryos to investigate the function of the transcription factor OCT4.^33^ OCT4 targeting led to decreased expression of trophectoderm and pluripotency genes, and blastocyst development was compromised, in line with other work.^29^ In addition, insulin growth factor 1 (IGF1) signaling maintains the ICM,^34^ whereas IGF1 treatment of human embryos promotes ICM proliferation.^34–36^ Whether other signaling pathways and transcription factors are involved in ICM specification remains unclear.

#### Second cell fate decision: Epiblast versus hypoblast

In the second cell fate decision, ICM cells segregate into epiblast and hypoblast cells. At this stage, the epiblast consists of naive pluripotent cells that serve as precursors of all fetal cells. Transcription factors SOX2, OCT4, and KLF17 are initially expressed in most cells of day 5 blastocysts, then become restricted to the epiblast by the late blastocyst stage (day 7).^16,27,37,38^ Naive epiblast cells are marked by the expression of core pluripotency factors NANOG, SOX2, and OCT4, as well as naive pluripotency-specific factors, such as TFCP2L1, PRDM14, KLF4, and KLF17.^16,27,30,33,37–41^ Key components of the transforming growth factor-β (TGF-β) signaling pathway are enriched in the human epiblast,^30^ although it remains unclear if this also applies to ICM cells. TGF-β signaling inhibition in human embryos abrogates NANOG expression,^30^ suggesting this pathway is required to specify the human ICM and naive pluripotent epiblast.

The hypoblast marker PDGFRA is initially expressed in all ICM cells, and its restriction to hypoblast cells is followed by expression of SOX17, FOXA2, and GATA4.^23^ Maintenance of hypoblast cells has been suggested to involve TEAD1 and YAP1.^22^ In the mouse, fibroblast growth factor (FGF)/mitogen-activated protein kinase (MAPK) signaling is important during primitive endoderm specification.^42^ Initial studies using human embryos and a MEK inhibitor reported that human hypoblast specification occurs independently of FGF/MAPK signaling.^38,43^ However, higher concentrations of the MEK inhibitor impair hypoblast specification in human embryos,^24,44^ suggesting the role of the FGF/MAPK signaling pathway is conserved across mice and humans.

#### Cell fate restriction and blastocyst maturation

In contrast to mice,^45,46^ lineages are not yet restricted in the human blastocyst. Human trophectoderm cells can still form ICM cells,^47^ and isolated ICM cells form trophectoderm cells upon dual MEK/NODAL inhibition.^3^ The molecular basis behind this difference in cellular plasticity between species remains unknown. Importantly, this variation in cell plasticity impacts the strategies used to design stem cell-based embryo models.

A molecular crosstalk between trophectoderm and epiblast is established as the blastocyst matures. Epiblast cells express transforming growth factor-β (TGF-β), insulin-like growth factor 1 (IGF1), bone morphogenetic protein 2 (BMP2), and fibroblast growth factor 4, whereas trophectoderm cells express platelet-derived growth factor (PDGF), interleukin-6 (IL-6), and WNT.^27^ Maturation of polar trophectoderm cells, in contact with the epiblast, is marked by the expression of NR2F2, which eventually spreads through the whole trophectoderm.^27^ However, the molecular basis of blastocyst maturation and the functional involvement of different candidate pathways remain to be investigated. As we will see in the following sections, their exploration can benefit from the use of stem cell-based embryo models.

### Human pre-implantation stem cell types

#### Naive PSCs

Naive human PSCs resemble the pre-implantation epiblast^48^ and are characterized by unrestricted developmental potential and lack of lineage biases.^49^ Naive human PSC lines can be established following resetting of primed human PSCs,^41,50,51^ by reprogramming somatic cells,^52^ or directly from human embryo ICM cells.^1^ Human naive PSCs form dome-shaped colonies, self-renew, and, importantly, differentiate into all three founding lineages of the human blastocyst. Due to their high developmental potential, naive human PSCs were thought to be devoid of epigenetic restriction. However, the Polycomb repressive complex 2 opposes induction of alternative cell fates in naive cells.^53,54^ It remains unclear if additional chromatin barriers to alternative cell fate induction exist in human naive PSCs.

**Figure 2:**
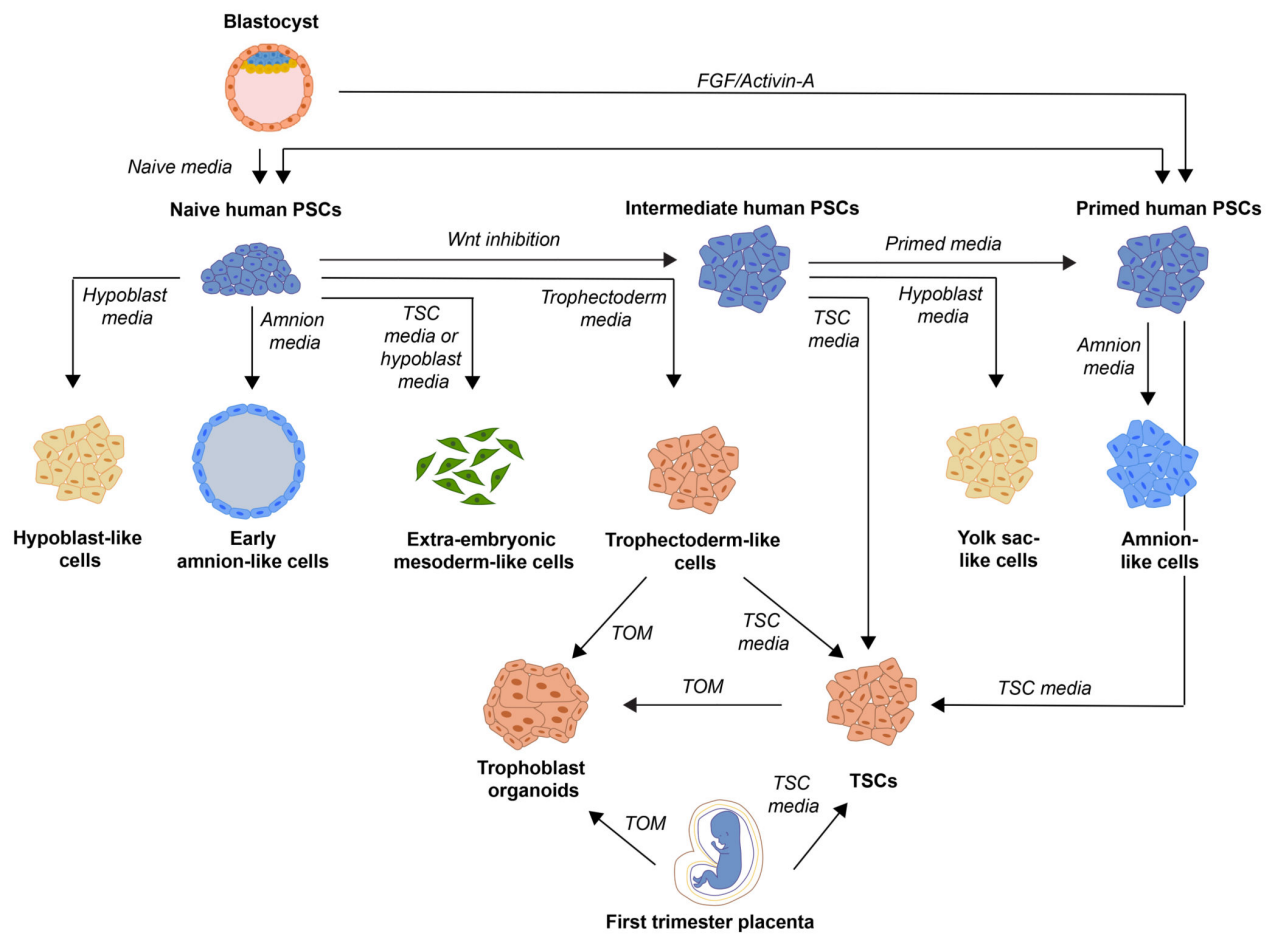
Stem cell types and transitions between them Cell types that can be derived from embryos, by reprogramming approaches or differentiation, which represent the founding cell types of the early human embryo. TOM, trophoblast organoid media; TSC, trophoblast stem cell.

Naive human PSCs have a transcriptome similar to the pre-implantation epiblast,^55,56^ including a hominoid-specific transposon profile.^57,58^ They also have low DNA methylation,^41^ two active X chromosomes,^57^ and a metabolism with activation of mitochondrial respiration.^41,56^ Concerns have been raised regarding the loss of imprinting and chromosomal instability in long-term human naive PSCs.^59^ More recent work has suggested that transient reprogramming to the naive state can erase the epigenetic memory of primed human-induced PSCs and partially safeguard imprints.^60^ Several culture conditions have been reported to derive and maintain human naive PSCs (Figure 2), yet there is no real consensus on the best conditions to use.

Human naive PSC cultures have also been used to model the eight-cell stage of human embryos, due to their intrinsic property to spontaneously give rise to a small proportion of eight-cell-like cells (8CLCs).^61–63^ However, eight-cell stage blastomeres and 8CLCs are distinct entities. Naive human PSCs and 8CLCs possess features of totipotent stem cells. However, it is not possible to test whether these cells are truly totipotent (Box 2).

Naive human PSCs are an excellent platform to model early human embryogenesis and can be used to study gene regulatory networks (GRNs) that are active in the pre-implantation epiblast. Transcription factors including TFCP2L1, KLF4, and NANOG are important for the maintenance of human naive pluripotency.^3,41^ Other transcription factors, such as KLF17, seem to be dispensable.^37^ In the future, naive human PSCs will help improve our understanding of the mechanisms of naive pluripotency and their genetic and epigenetic regulators and allow comparisons between species.

#### Trophectoderm stem cells

Upon dual MEK and NODAL inhibition, naive human PSCs form trophectoderm-like cells, which express the trophectoderm transcription factor CDX2^68^ but lack expression of sialic acid-binding Ig-like lectin 6 (SIGLEC6), which is expressed in post-implantation trophoblasts.^4^ However, these trophectoderm-like cells cannot be maintained long-term in culture.^3,4^ A recent publication has reported the derivation of CDX2+ trophectoderm-like cells from primed human PSCs. However, the transcriptional profile of these cells has not been compared with the embryo.^69^ Therefore, stable trophectoderm stem cell lines remain to be derived from the embryo or other sources.

#### Hypoblast stem cells

Our current knowledge of hypoblast development and maturation is limited, despite its relevance as a major signaling center during early post-implantation development. Comparisons between embryonic endoderm and extra-embryonic hypoblast have identified a set of markers, including PDGFRA, APOA1, RSPO3, HNF4A, LGALS2, and NID2, that are expressed in extra-embryonic but not embryonic endoderm.^44,70^ Using naive human PSCs as a starting point, hypoblast-like cells have been obtained *in vitro* by concurrent ACTIVIN-A, WNT, and JAK-STAT pathway activation,^5^ although their pre-implantation characteristics have been questioned.^71^ Supporting this approach to generate hypoblast cells, inhibition of ACTIVIN-A decreases the number of epiblast and hypoblast cells in human embryos,^30^ and WNT activation is required for hypoblast specification in marmoset embryos.^72^ An alternative route to form hypoblast cells entails a pre-treatment of naive human PSCs with MEK and NODAL inhibitors to obtain an ICM-like state, which responds to FGF and WNT/NODAL inhibition by forming hypoblast cells.^44^ More recently, human hypoblast-like cells have been obtained by activation of the FGF and BMP pathways together with WNT/NODAL inhibition.^71^ This treatment also leads to hypoblast specification from monkey ICMs, but derivations from human blastocysts have not been achieved yet.

## Implantation and Post-Implantation Development

The embryo is ready to implant into the maternal uterus when it reaches the late blastocyst stage and hatches from the *zona pellucida* by day 7 post-fertilization. Embryo implantation begins with the fusion of polar trophectoderm cells to form a primary syncytium, which penetrates the endometrial epithelium and invades the underlying stroma.^73,74^ Implantation involves a complex dialogue between a competent embryo and a receptive endometrium. Subtle alterations of this crosstalk can have fatal consequences, accounting for approximately 30% of pregnancy losses.^75^ This developmental period has been particularly challenging to study, given the inaccessibility of the implanting human embryo. As an alternative to *in vivo* studies, several *in vitro* models of implantation have been developed.^76^ Co-culture between primary human endometrial epithelial cells and human embryos was established to study embryo apposition and attachment, the initial phases of implantation.^77^ In this setting, the subsequent phase of implantation, invasion, was not recapitulated. Invasion can be modeled *in vitro* by placing hatched human blastocysts on top of a monolayer of primary endometrial stromal cells.^78,79^ More complex models include both endometrial epithelial and stromal cells,^80,81^ although they cannot be maintained long-term. The subsequent development of hormone-responsive endometrial organoids overcame this limitation. Endometrial organoids mimic the molecular and histological features of the epithelial compartment of the endometrium.^82,83^ Endometrial assembloids, including both epithelial and stromal cells, have also been reported,^84^ but currently they do not support proper post-implantation morphogenesis.

To date, studies have combined endometrial organoids and blastoids to study implantation. Culturing endometrial organoids as monolayers allows the interaction and attachment of blastoids only if the endometrial cells are hormonally stimulated.^9^ Interestingly, when trophospheres devoid of an ICM are plated on top of the hormonally stimulated endometrial cells, attachment does not take place. This observation indicates that the ICM endows polar trophectoderm cells with the potential to initiate implantation.^9^ Although this system represents a physiologically relevant model to study the initial steps of implantation, the generation of an *in vitro* model of implantation that recapitulates successful post-implantation morphogenesis is still a fundamental challenge in the field. Such an *in vitro* model would help us explore the mechanisms of implantation, dissect the contribution of the endometrium to embryo morphogenesis, and determine what goes wrong when a successful pregnancy is not established.

### From blastocyst to gastrula

Upon implantation, the human blastocyst initiates a phase of rapid proliferation and major reorganization to form the basis of the body plan and associated extra-embryonic membranes. However, studying the early post-implantation human embryo is very challenging, as *in vivo* studies are not possible for ethical and technical reasons, and human embryos are typically cultured *in vitro* only up to the blastocyst stage.^85^ In the next sections we describe our current knowledge of human embryo development after implantation in the uterus *in vivo* and upon extended culture *in vitro*.

#### *In vivo* embryo development

During the last century, thousands of embryos developing *in vivo* were collected after hysterectomy and analyzed by electron microscopy.^26^ These studies led to the creation of a morphological atlas of early post-implantation human development.^86,87^ Pregnancy terminations represent another potential source of human embryos for scientific research, but these are typically carried out at later developmental stages.^88^ However, it was possible to analyze a human embryo, obtained from a pregnancy termination, at the single-cell level 16–19 days after fertilization.^89^ This embryo represents a rare transcriptional reference of an *in vivo* gastrulating human embryo. Access to more precious samples at such early stages will be fundamental in creating an accurate molecular atlas of *in vivo* developing human embryos. Such a reference atlas is of utmost importance to benchmark stem cell-based embryo models, as we discuss in the next section.

Electron microscopy images have demonstrated that upon implantation, the epiblast undergoes a process of epithelialization and concomitant fate split. Epiblast cells in contact with the trophoblast differentiate to form squamous amniotic epithelial cells, the amnion, whereas epiblast cells in contact with the hypoblast form the pluripotent epiblast disc, a pseudostratified epithelial tissue (Figure 1).^90^ The post-implantation epiblast is an apicobasal, polarized tissue that has lost its naive character and transitioned into an intermediate state, also referred to as formative, pluripotent state.^91^ By day 8–9 post-conception, epiblast and amnion are contiguous and enclose the amniotic cavity, which has a protective role during embryo development.^90,92^ Hypoblast cells give rise to the primary yolk sac around day 7–12, which is replaced by a secondary, definitive yolk sac by day 14, at the onset of gastrulation.^25^ This definitive yolk sac is the first site of hematopoiesis and mediates nutrient exchange, which is especially important during the first weeks of post-implantation development when the placenta is not well vascularized.

Trophectoderm cells give rise to cytotrophoblast (CT) cells, the stem cell population of the placenta, which proliferate and differentiate to form placental villi, the basic functional unit of the placenta.^93^ CT cell fusion leads to the formation of multinucleated syncytiotrophoblast (SCT) cells, which secrete pregnancy hormones, such as human chorionic gonadotropin. Fusion is mediated by human endogenous retroviral proteins, such as SYNCYTIN-1.^94^ In addition, a subset of villous CT cells proliferate to form columnar CT, a highly proliferative progenitor population that upon epithelial to mesenchymal transition (EMT) forms migratory extravillous trophoblast (EVT) cells, which play a fundamental role in the formation of the fetal-maternal interface.^95^

The extra-embryonic mesoderm (ExM) is another cell type that becomes specified during early post-implantation development, starting around day 11, from unknown origins.^96–99^ ExM cells migrate to multiple locations in the conceptus, lining the inner surface of the CT, supporting the epithelium of the amnion and yolk sac, filling trophoblast villi, and forming a stalk region that connects the embryo to the CT and acts as the primary umbilical cord (Figure 1). ExM cells go on to form the first blood cells of the developing embryo. Primordial germ cells (PGCs), the progenitors of the gametes, also become specified shortly after implantation, although the specific timing is unclear.^100^ The origin of human PGCs remains a matter of debate. In cynomolgus monkey and marmoset embryos, PGCs arise from the amnion,^101,102^ whereas in porcine embryos, PGCs develop from posterior epiblast cells.^103^ In humans, a dual origin is considered a possibility.^104^

#### *In vitro* embryo development

Human embryos have been cultured *in vitro* beyond the blastocyst stage.^39,105^ In the absence of maternal tissues, they attach to the dish and undergo some of the landmarks of early post-implantation morphogenesis, including amniotic cavitation, primary yolk sac formation, and trophoblast differentiation.^39,105^ This initial methodology has been broadly employed and further refined. As a result, *in vitro* developing human embryos have been extensively characterized at the morphological, genomic, transcriptional, and epigenetic level.^40,106–111^

Upon *in vitro* culture of human blastocysts, epiblast cells exit from the naive pluripotent state, upregulate the expression of post-implantation factors, switch their metabolism from oxidative phosphorylation to glycolysis, gain DNA methylation, and initiate the process of random X-chromosome inactivation.^106,111,112^ A subset of epiblast cells differentiates to make the amnion,^40^ and ExM cells appear at the border between the epiblast, hypoblast, amnion, and trophoblast. ExM cells display an EMT signature, and their specification requires dual activation of WNT and BMP.^111^ ExM cells express high levels of BMP2 and BMP4, whereas amnion cells express the BMP targets ID1 to ID4, suggesting intercellular communication between these two cell types. The origin of ExM cells remains unclear,^99^ with potential epiblast,^99,111^ hypoblast,^97,113,114^ trophoblast, or a combination of origins,^26^ and further exploration is needed.

In the hypoblast, a subset of cells that expresses inhibitors of WNT, BMP, and NODAL signaling become asymmetrically distributed.^108^ These cells require NODAL signaling for their specification and BMP for their maintenance.^115^ They have been proposed to represent an anterior signaling center, similar to the anterior visceral endoderm (AVE) of the mouse embryo,^116^ that could be involved in setting up the anterior-posterior axis. In agreement, by day 12 of *in vitro* culture, BRACHYURY becomes asymmetrically localized within the epiblast cluster,^117^ but embryos do not show proper morphological organization. Therefore, additional studies are needed to demonstrate the role of the human AVE in setting up the anterior-posterior axis. PGCs have also been identified in day 12 *in vitro* cultured human embryos,^118,119^ indicating the inductive cues necessary for their specification are recapitulated in a small proportion of embryos *in vitro*. Lineage-tracing studies may shed light on their tissue of origin and the mechanisms that control the soma-germline bifurcation.

The study of human embryos cultured *in vitro* raises the important question of how the uterus contributes to embryo morphogenesis. With current *in vitro* culture methodologies, embryos partially initiate early post-implantation morphogenesis, but the efficiency of development progressively decreases up to day 13. Is the uterus needed to sustain the more complex morphogenetic events that take place at the onset of gastrulation? In mouse embryos, there is conflicting evidence as to whether physical forces arising from the uterus modulate AVE specification.^120,121^ *In vitro* development of mouse embryos can be achieved from early post-implantation (E5.5) to late organogenesis (E11).^122^ However, the embryos used at the beginning of the experiment had already implanted *in vivo*, and this initial interaction with the uterus could be essential for their subsequent *in vitro* development. Conversely, monkey embryos created *in vitro* have been cultured from the zygote stage all the way to an early organogenesis stage.^123–125^ In human embryos, successful placentation and trophoblast differentiation require close interactions with uterine cells.^126–128^ Overall, how important these events are for instructing the organization of the embryo it-self is not yet clear.

Although they are data-rich and informative, studies using *in vitro* cultured human embryos mostly remain descriptive, as it is challenging to dissect molecular mechanisms. A report studying the development of embryos harboring chromosomal alterations demonstrated that human embryos cultured *in vitro* can be used to determine developmental competency.^129^ Therefore, one could envision the combination of CRISPR-Cas9 genome editing techniques and *in vitro* culture methodologies to examine gene function during human development. However, these studies will be limited by the scarcity and variability of donated human embryos, as well as by the current ethical and technical limits for the *in vitro* culture of human embryos.^85^ For this reason, stem cell lines and stem cell-based embryo models have emerged as promising genetically tractable alternatives.

### Human post-implantation stem cell types

#### Epiblast and derivatives

Conventional human PSCs are epithelial, require ACTIVIN-A and FGF signaling for their maintenance,^2,130,131^ and are molecularly similar to the early gastrulating epiblast.^89,108,113^ They also possess a GRN governed by NANOG, OCT4, and SOX2,^132^ and display features of primed pluripotency. In addition, an intermediate pluripotent state, the formative state, has been confirmed as a state of competency for germline and somatic differentiation.^91,133,134^ This finding is based on the observation that mouse epiblast cells are responsive to differentiation cues during a short developmental window, between implantation and gastrulation, and hence between the naive and primed states.^135,136^ During human embryo development, a 7-day-long timespan between the naive (days 6–7) and primed (days 13–14) pluripotent state supports the existence of an intermediate pluripotent state. Formative human PSCs are maintained either in a media containing low levels of ACTIVIN-A, a retinoic acid receptor agonist, and a WNT inhibitor^134^ or under FGF, TGF-β, and WNT signaling activation.^137^ Although epiblast cells in early post-implantation embryos (prior to day 14) clearly exhibit molecular features that are intermediate between naive and primed pluripotency, the concept of human formative pluripotency as a unique functional entity remains contentious, as human PGCs can be induced from primed PSCs.^138^

Lately, some of the cells that were thought to be naive^139^ or to present an expanded pluripotent potential,^140^ have been shown to be transcriptionally similar to the early post-implantation epiblast and thus fall under the category of intermediate pluripotency.^52,137^ To what extent these intermediate pluripotent cells are similar to formative human PSCs has not been investigated. Moving forward, it will be important to carefully compare different pluripotent cultures at the molecular level (epigenome, transcriptome, and proteome), as the initial pluripotent state of the cells determines their capacity to form extra-embryonic cells, and hence to model different stages of human embryogenesis.^9,11,141^ For example, when primed and formative PSCs are used as a starting point to generate blastoids, the resulting structures contain off-target cell types, such as amnion and trophoblast analogs akin to post-implantation stages.^141,142^

The first tissue that becomes specified from epiblast cells *in vivo* is the amnion. The generation of amnion-like cells *in vitro* has been achieved.^143^ When primed human PSCs are cultured in a 3D gel of extracellular matrix proteins, they form amniotic spheroids in a BMP-dependent manner.^144^ In 2D, culture of human PSCs in micropatterns in the presence of BMP4^145^ leads to the emergence of amnion-like cells in the outermost region of the colonies.^146^ Directed differentiation protocols involve the addition of BMP together with NODAL and MEK inhibitors to the medium,^3,4^ but whether the resulting amnion-like cells can be maintained long-term has not been explored. Given that the amnion appears shortly after implantation and human primed PSCs represent the early gastrulating epiblast, their capacity to generate amnion-like cells is surprising. Pseudotime analyses revealed that early post-implantation epiblast cells give rise to early amnion cells, which express *GATA2, GATA3*, and *TFAP2A* and are transcriptionally similar to trophectoderm cells,^143^ whereas early gastrulating cells give rise to late amnion cells that express *GABRP* and share transcriptional similarity with the non-neural ectoderm.^143^ However, early amnion cells are not found in *in vitro* cultured gastrulating cynomolgus macaque embryos,^147^ and thus their final fate is unknown, and a progressive maturation of amnion cell from an early to a late state cannot be ruled out.^10,111,148^

#### Yolk sac cells

The molecular differences between pre- and post-implantation hypoblast remain contentious. Post-implantation yolk sac-like cells have been derived from intermediate PSCs.^70^ Initial studies proposed that naive PSCs form pre-implantation hypoblast-like cells.^5^ However, later studies reported that the resulting cells reflect post-implantation stages.^44,71,99^ Overexpression of GATA6 or SOX17 in primed human PSCs is an alternative strategy to induce a yolk sac fate.^149,150^ However, the resulting cells cannot be maintained long-term, and their gene expression profile has not been compared with the embryo. Lastly, combining stromal cells and primed PSCs in the absence of any external morphogens leads to the formation of yolk sac-like organoids that contain hematopoietic progenitor cells.^151^ Therefore, under specific *in vitro* culture conditions, primed pluripotent cells might regain the ability to form post-implantation hypoblast derivatives.

#### ExM cells

Naive human PSCs grown in trophoblast stem cell (TSC) media generate not only TSCs but also ExM cells.^99^ Stem cell-derived ExM cells express markers associated with hypoblast, amnion, mesoderm, and other cell types, but in a combination unique to the *in vivo* ExM.^111^ The BMP4 and mTOR pathways have been implicated in ExM cell maintenance in humans and monkeys,^99,152^ while WNT and BMP inhibition reduce ExM specification.^111^ Differentiating naive human PSCs toward the hypoblast lineage can also give rise to ExM cells, with pathway modulation influencing cell outcomes.^12,99,111^ The exact reasons for ExM cell emergence during these processes are not fully understood.

#### PGCs

The human epiblast also transitions through a phase of competency for germline entry. This discovery was important for the first protocols that describe the generation of PGC-like cells *in vitro*. Intermediate PSCs form PGC-like cells upon aggregation and exposure to BMP.^153^ Accordingly, cells become competent for PGC specification 2 days after removal of naive factors from the media.^154^ Alternatively, if primed human PSCs are differentiated into incipient mesoderm-like cells, they gain competency for PGC-like cell specification.^138^ In this context, competent cells exhibit active NODAL and WNT signaling, downregulate OTX2, and express mesoderm-related genes such as EOMES.^155^ Interestingly, the mechanisms of PGC specification vary depending on the initial precursor.^154^ Understanding the active mechanisms in the human embryo and the cell of origin of human PGCs is therefore essential.

#### Trophoblast and derivatives

A set of criteria define TSCs, namely self-renewal, expression of trophoblast markers, such as GATA3 and TFAP2C, a unique pattern of expression of human leukocyte antigen (HLA) molecules, hypomethylation of the promoter of the trophoblast gene *ELF5*, and high levels of expression of the C19MC micro-RNA cluster.^17^ TSCs that meet all the criteria have been derived from human blastocysts and first-trimester placentas^156^ using MAPK and WNT activators together with TGF-β/NODAL, histone deacetylase (HDAC), and ROCK inhibitors.^156^ Under these conditions, TSCs are bipotent and able to differentiate into EVT and SCT. However, their *in vivo* counterpart remains unclear. When 2D TSCs are compared with pre- and early post-implantation *in vitro* cultured embryos, they transcriptionally resemble the peri-implantation trophoblast,^68,110^ but when they are compared with first-trimester placentas, they resemble columnar CT, in agreement with their EVT differentiation bias.^157^

3D cultures of human TSCs have also been reported.^158,159^ These trophoblast organoids are derived from first-trimester placentas and contain a layer of CT cells that spontaneously differentiates to form a core of SCT, recapitulating an inverted villous architecture. They can also give rise to EVT when WNT activators are removed from the media. Trophoblast organoids capture villous CT cells,^157,160^ which generate SCT upon cell-cell fusion. Therefore, 2D TSCs and trophoblast organoids capture different progenitor populations.

TSCs may be generated by exposing intermediate PSCs to BMP^161^ or naive human PSCs to TSC medium.^68,161,162^ Likewise, naive human PSCs can be a starting point to generate trophoblast organoids.^68,163^ The derivation of TSCs from primed human PSCs has been a topic of debate, and the resulting cells have been proposed to represent amnion-like cells.^3,4,146^ However, by performing a pre-induction step to inhibit TGF-β/NODAL and WNT signaling prior to exposure to TSC medium, primed human PSCs differentiate into TSCs that are transcriptionally and epigenetically similar to bona fide TSCs.^164,165^ Intriguingly, in doing so, primed human PSCs transiently upregulate amnion markers, which could suggest the initiation of an amnion-like program in the route toward trophoblast.^164^ TSCs can also be induced directly from fibroblast by reprogramming without a pluripotent intermediate.^166,167^ To what extent these findings are relevant in an embryo context remains unknown, but they extend the cell types, differentiation, and reprogramming protocols that can be applied to study the trophoblast and model embryo development. Careful comparisons between the different TSC lines and the *in vivo* placenta are still needed to map *in vitro* stem cells lines to their *in vivo* counterpart.

The quality of the stem cell lines is a major determinant of the success of embryo model generation. A careful characterization of the molecular and functional characteristics of these cells should be undertaken prior to generating complex structures. However, this validation comes with its own limitations, as *in vivo* reference datasets are limited, and functional assays are technically challenging. Studying cell types in isolation is also important to identify cell autonomous mechanisms of cell behavior. For biological questions that, for instance, touch on fate decisions of individual cells or the identification of the GRN of a particular cell type, it might not be necessary to use complex embryo models. For other questions pertaining to tissue crosstalk, more complex models become indispensable.

## Stem Cell-Based Embryo Models

**Figure 3:**
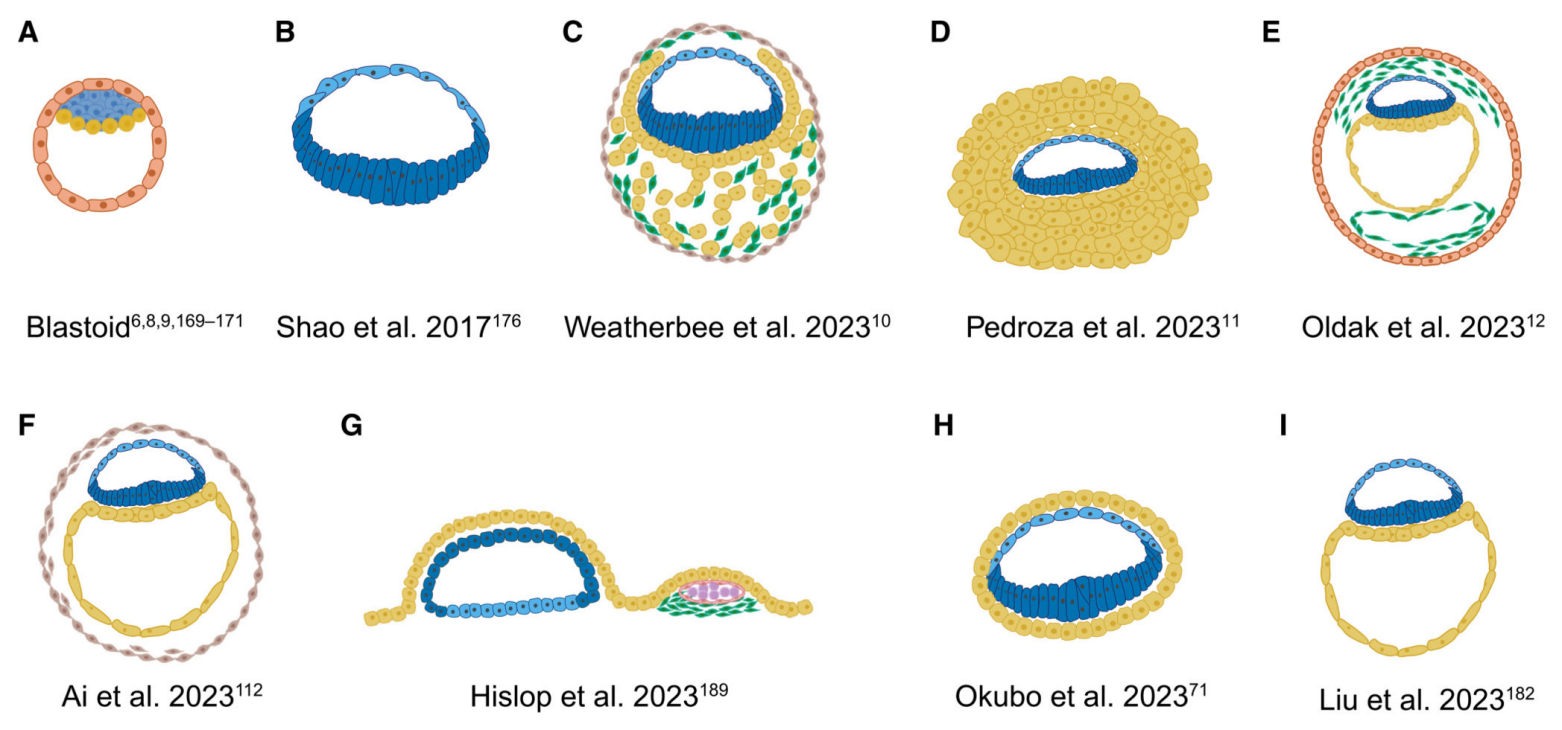
Stem cell-based models of the human embryo Overview of published stem cell-based human embryo models. Blastoids have been reported by Yu et al.,^6^ Yanagida et al.,^8^ Kagawa et al.,^9^ Guo et al.,^168^ Yu et al.,^169^ and Karvas et al.^170^ Epiblast and amnion are shown in blue, hypoblast and yolk sac in yellow, trophoblast in orange, extra-embryonic mesoderm in green, extra-embryonic cells of unknown identity in light brown, and hematopoietic stem cells in pink.

Over the years, several human embryo models have been developed (Figure 3). Here, we have classified these models based on the starting cell type and the developmental stage they mimic.

### Pre-implantation models

Human naive PSCs can self-organize into structures, termed blastoids, that adopt a morphology similar to human blastocysts and contain cells of the three founding lineages.^6,8,9^ Because blastoids can be generated at scale and from different genetic backgrounds, they are predicted to support medical advances through the understanding of early human embryogenesis and the improvement of ART.

Several blastoid models have been developed. In general, about 50 naive human PSCs are grown in microwells in the presence of HIPPO, TGF-β, and MEK inhibitors and leukemia inhibitory factor (LIF), which trigger cavity formation and lineage specification.^9^ A study has also reported the “spontaneous” formation of human blastoids from naive PSCs.^168^ Excitingly, blastoids form cells of the three founding lineages in average ratios approaching those of blastocysts, and their transcriptome is similar to the blastocyst.^9,169^ Blastoids break symmetry and induce an embryonic-abembryonic axis with epiblast analogs located on one side. Similar to human blastocysts,^20^ trophectoderm specification and morphogenesis within blastoids depends on aPKC signaling and inhibition of the HIPPO pathway.^6,9^ Importantly, the correct sequence and pace of lineage specification can be recapitulated in blastoids.^9^ Specifically, trophectoderm fate induction, marked by GATA3 and GATA2 expression, takes place before hypoblast fate specification, marked by GATA4 and SOX17 expression, and blastoid formation is completed within 4 days. In late blastoids, polar and mural trophectoderm cells become specified, and cycles of cavity expansion and collapse are observed similar to blastocysts.^6,9^

Studies using blastoids have started to investigate the role of signaling in cell fate specification, including tissue crosstalk. For example, the maturation of polar trophectoderm cells depends on signals from epiblast cells, and this allows attachment of trophectoderm cells to the endometrium.^9^ Phosphatidylinositol 3-kinase (PI3K)/AKT, mTOR, and AMPK pathways are necessary for human blastoid derivation,^169^ and hypoblast formation requires FGF/MAPK signaling.^24,44^ Future studies will enable the exploration of cell fate decisions, morphogenesis, and epigenetic regulatory mechanisms, such as X-chromosome inactivation, DNA methylation, and transposon biology, providing insights into the mechanisms of early human embryogenesis. Blastoids can also form in human embryo culture conditions^171^ and therefore may be used to improve media formulations, which could have clinical impact. Although blastoids represent a more ethical alternative, they are not a replacement for the use of human embryos in research.

### Bridging pre- and post-implantation development

Blastoids represent an ideal model to bridge the pre- and post-implantation transition *in vitro*. Initially, blastoids were grown on 2D plastic plates or with hormonally stimulated endometrium or-ganoid-derived cells.^6,7,9^ Within a few days, blastoids specified the SCT and EVT lineages, expressed clinical pregnancy levels of human chorionic gonadotropin, showed epiblast polarization, and formed amniotic-like cavities at low efficiency.^6,7,9^ However, blastoids did not recapitulate the cell type composition and organization or later developmental stages equivalent to day 13 embryos. Therefore, there is a need to improve the post-implantation culture protocols to better model *in vivo* development. A later study showed that blastoids cultured on endometrial stromal cells grow more and have reduced apoptosis when compared with blastoids cultured on fibronectin.^169^ Immortalized endometrial stromal cells promoted the proliferation of epiblast-like and TE-like cells and trophoblast syncytialization in blastoids and blastocysts.

Interestingly, blastoids grown in 3D matrices capture several hallmarks of early post-implantation embryogenesis, including epiblast luminogenesis, diversification of trophoblast lineages, and robust invasion of EVT cells by 14 days of culture.^169,170^ However, compared with embryos, blastoids show delayed development. By day 14, blastoids lack several cell types (i.e., ExM, primitive streak, and amnion) and do not exhibit the correct morphology. Extended culture (day 21) results in ExM specification and expansion, localized activation of the primitive streak marker BRACHYURY, and the emergence of embryonic germ layers as well as PGC-like cells (PGCLCs), in line with a developmental delay. Despite these advances, the morphology and cell organization of blastoids grown to post-implantation stages in 3D are significantly different from that of natural embryos.

### Post-implantation models

A common strategy to build embryo models from stem cells is to generate 3D aggregates of PSCs in suspension. When intermediate pluripotent cells are grown as small aggregates, they form structures encompassing an outside layer of yolk sac-like cells that surrounds the epiblast-like and amnion-like compartments.^11^ These structures cannot be formed from primed cells, in agreement with the lack of hypoblast competency of primed cells previously reported.^5,70,71^ An AVE-like domain is present in the yolk sac-like compartment, which antagonizes amnion formation and potentially mesoderm specification as well.^70^ Although this study exploited the self-organizing capacity of PSCs, another alternative is based on the use of transcription factor overexpression to guide cell fate specification. Overexpression of transcription factors GATA6/SOX17 and GATA3/AP2γ in intermediate PSCs is sufficient to generate hypoblast- and trophoblast-like cells, respectively.^10,172^ These two cell types are then combined with intermediate pluripotent cells, as representative of the post-implantation epiblast. The resulting structures contain amnion-like cells, ExM-like cells, and PGCLCs, but the GATA3/AP2γ overexpressing cells upregulate GATA6, indicating that they do not represent bona fide trophoblast. Interestingly, the authors found that SOX17 overexpression inhibits AVE specification, and therefore the high levels of BMP signaling present in the structures lead to epiblast differentiation.

Human naive PSCs have also been combined with extra-embryonic-like stem cells to generate complex embryo models. One study has reported the formation of assembloids, made by combining human naive PSCs and extra-embryonic cells, which act as a signaling nest for the embryonic compartment.^111^ The authors used analyses of *in vitro* cultured human embryos to fine-tune the activation of key signaling pathways, such as BMP, WNT, and NODAL/ACTIVIN-A. This step was followed by self-organization in the absence of exogenous cues, leading to the formation of a stem cell-based embryo model containing an epiblast disc, amniotic, and yolk sac-like cavities, PGCLCs, and ExM-like cells, which in this system are derived from epiblast-like cells.^111^ This is a good example of how embryo studies can help rationally design protocols to improve stem cell-based embryo models.

Another study has combined embryonic and hypoblast-like cells to generate a bilaminar structure composed of a pluripotent epiblast and an amnion-like epithelium that is surrounded by yolk sac-like cells.^71^ The resulting “bilaminoids” can develop to mimic the beginning of gastrulation. The utility of this model to study intercellular communication has already been demonstrated by adding TSCs using a transwell assay. TSCs secrete IL-6, which causes epiblast proliferation and amniotic cavitation.^71^ Lastly, a model of the post-implantation human embryo has been obtained by combining epiblast-, hypoblast-, ExM, and trophoblast-like cells, which were generated from naive human PSCs.^12^ The resulting structures contain an epiblast disc, amniotic and yolk sac-like cavities, ExM-like cells, and PGCLCs. Moreover, they break the anterior-posterior symmetry and display AVE and primitive streak-like domains.^12^ This model recapitulates key hallmarks of post-implantation human embryos but also has some limitations, such as the presence of an underdeveloped trophoblast and the incomplete spatial organization of the ExM. The low efficiency of the protocol will also need to be improved to facilitate functional studies to dissect the mechanisms of development.

A common theme in post-implantation models is the lack of a bona fide trophoblast compartment, which in some cases leads to an unnatural direct interaction between the amnion and the yolk sac.^10,11^ In fact, the reported amnion-like cells fail to develop a convincing squamous epithelial morphology in most of the models, and this could be due to the lack of trophoblast-derived tissues. In some of the models, the function of the trophoblast may be partially replaced by the exogenous signals that are added to the medium and/or the physicochemical cues provided by other extra-embryonic cell types. Clarifying the contributions and interactions of exogenous and endogenous factors as well as different extra-embryonic tissues is an important area for future investigation. Adopting a modular approach to solve this question has some clear advantages, including the possibility of manipulating genes in specific tissues and altering specific components.

When attempting to recapitulate events that take place during gastrulation, primed human PSCs are the ideal starting point. Human PSCs cultured in a 3D gel of extracellular matrix proteins become apicobasally polarized and form a central lumen.^105,173,174^ Interestingly, the fate of these epithelial spheroids is in part controlled by density. Higher densities preserve a pluripotent identity, whereas lower densities lead to amnion specification in a BMP-dependent manner.^144,175^ Intermediate densities lead to symmetry breaking in approximately 5% of structures; one side of the spheroid remains pluripotent and initiates an EMT-like event, whereas the other acquires an amnion-like identity.^175^ This spontaneous symmetry-breaking event can be controlled. Directional exposure of spheroids to BMP4 leads to the formation of post-implantation human amniotic sacs.^176^ This robust and highly controllable model reveals that amnion-like cells trigger gastrulation-like events in the pluripotent compartment, including specification of mesoderm and PGCLCs.^176^ In the mouse embryo, the trophectoderm-derived extra-embryonic ectoderm acts as the main source of BMP during early post-implantation development,^177^ but human embryos do not have an extra-embryonic ectoderm compartment. Therefore, this study raises the interesting possibility that in humans, the amnion has replaced the signaling function of the extra-embryonic ectoderm during gastrulation. The ExM may act as an additional source of BMP.^99^ Addition of BMP4 to pluripotent epithelial spheroids has also been reported to break anterior-posterior symmetry and induce primitive streak fates in a WNT-dependent manner.^178^ Interestingly, such diverse outcomes of BMP stimulation, amnion versus primitive streak fates, could be controlled by the duration rather than the concentration of BMP.^179^ Globally, these 3D models represent an ideal platform to study the coordination between cell and tissue organization and cell fate decisions.^90^ They will likely be able to provide important insights into questions about the influence of the physical environment on cell identity and intercellular communication.

3D aggregates of primed and expanded potential human PSCs also generate more complex embryo models. In the case of primed cells, by sequentially providing a hypoblast induction media followed by an amnion induction media, structures containing an epiblast disc, amniotic- and yolk sac-like cavities, PGCLCs, and ExM-like cells have been reported.^180^ In this model and based on pseudotime analyses, PGCLCs are derived from posterior epiblast cells, whereas ExM-like cells are derived from the hypoblast.^180^ This work supports the idea that primed PSCs are competent to form hypoblast-like cells, although this capability has not been functionally evaluated. Alternatively, when expanded potential stem cells are exposed to FGF, TGF-β, and WNT activation, they generate aggregates that contain epiblast- and hypoblast-like domains. These aggregates recapitulate amniotic and yolk sac cavity formation, PGCLC specification, and aspects of primitive streak formation, gastrulation, and organogenesis.^181^ It remains to be explored whether the starting pluripotent state of the cells and their interactions with the exogenous factors affect the outcome of the model.

When primed human PSCs are grown in confined environments, they recreate the spatial patterning of the gastrulating human embryo.^145^ Likewise, growing them as embryoid bodies in the presence of a WNT agonist recapitulates the spatiotemporal organization of cell fates across the anterior-posterior axis in the absence of extra-embryonic cells.^182^ These so-called 2D and 3D gastruloids mimic post-gastrulation stages of development and hence will not be covered in this review, but we refer the reader to excellent publications elsewhere.^183–187^

**Figure 4:**
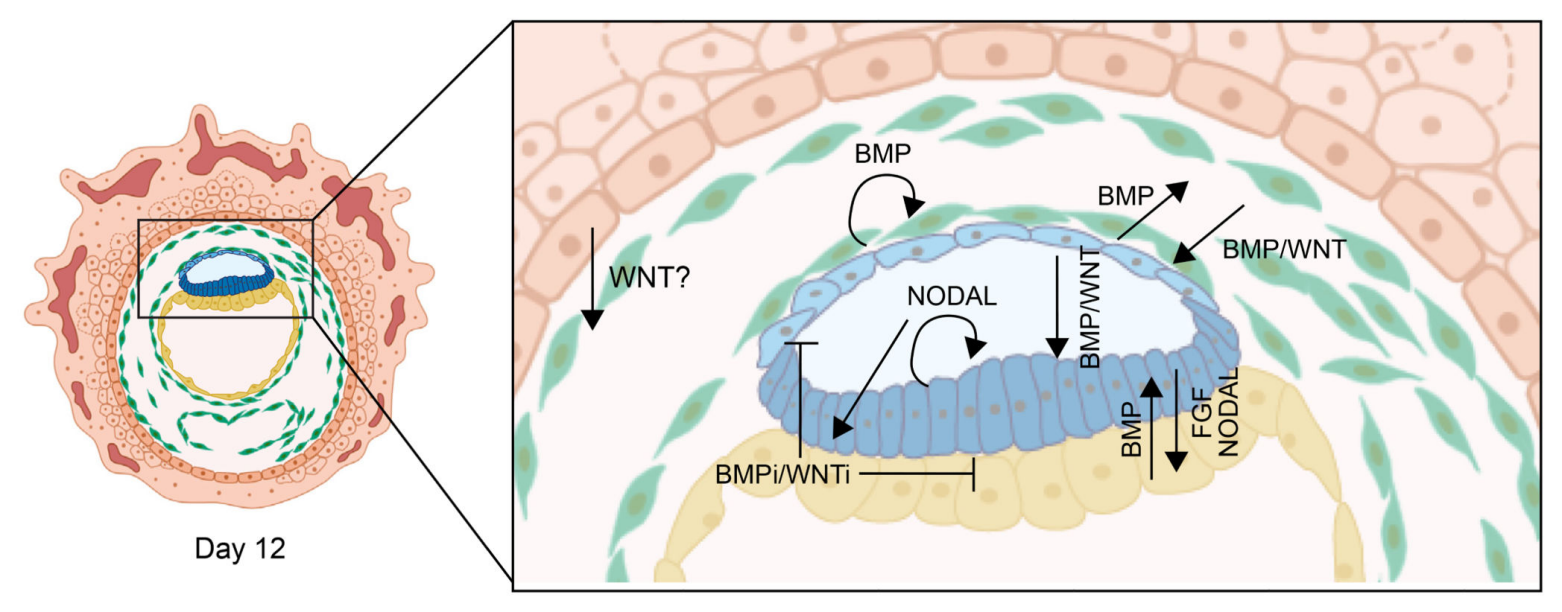
Intercellular interactions and signaling across cell types in the early post-implantation human embryo Signaling crosstalk that has been identified based on the *in vitro* culture of human embryos and the study of stem cell-based models is highlighted. Amnion, extra-embryonic mesoderm (ExM), and yolk sac cells are sources of BMP and WNT signals.^71,99,111,175,176^ WNT is potentially also secreted by cytotrophoblast (CT) cells.^190^ NODAL is secreted by the post-implantation epiblast and acts in an autocrine and a paracrine manner. A putative anterior signaling center is formed in the yolk sac in a NODAL-dependent manner.^71,115^ This anterior signaling center is a source of BMP and WNT inhibitors. Epiblast-derived FGF is needed for hypoblast specification.^44,108,111^ Blue marks epiblast and amnion, yellow marks hypoblast and yolk sac, green marks ExM, and orange marks the trophoblast.

To model gastrulation, two or more cell types have also been combined. These cell types need to self-assemble to recapitulate intercellular interactions and generate an embryo-like morphology. Combining yolk sac-like cells together with PSCs in a 3D gel demonstrated that yolk sac-like cells prevent differentiation of PSCs into mesoderm and endoderm by blocking BMP and WNT signaling.^70^ However, an embryo-like architecture was not observed. This limitation was solved by co-culturing human PSCs, as representative of the post-implantation epiblast, and GATA6 overexpressing PSCs, to mimic the yolk sac, in 2D.^188^ Under these conditions, human PSCs formed asymmetric epithelial spheroids; cells in contact with the culture dish differentiated to form amnion-like cells, and cells in contact with the yolk sac-like compartment retained an epiblast identity. In agreement with previous results,^70^ the yolk sac-like cells expressed BMP and WNT inhibitors and were potentially in control of breaking the anterior-posterior symmetry (Figure 4). Moreover, in this model, the yolk sac compartment matured to allow hematopoiesis.^188^ Embryo-like structures have also been obtained by combining extra-embryonic-like stem cells, whose identity and embryonic counterpart remain unclear, with PSC-derived spheroids.^189^ Upon attachment and culture in post-implantation human embryo media,^39,105^ these assembloids break anterior-posterior symmetry and develop asymmetric BRACHYURY/SOX2 expression.^178^ Globally, these complex 3D models are very well suited to study cell fate specification events, intercellular communication, and the mechanisms of self-organization during gastrulation.

## Current Limitations of Stem Cell-Based Embryo Models

Even though stem cell-based embryo models promise to transform the study of human embryogenesis, there are several limitations that need to be overcome before these models can be broadly applied.

### Limitations of pre-implantation models

Blastoids do not model the stages that precede the blastocyst stage, which, importantly, include the early cleavage and morula stages that are especially susceptible to failure. Several blastoid models generate lineages according to the pace and sequence of blastocyst development,^8,9^ whereas others take longer and induce hypoblast cell specification before trophectoderm induction.^6,169,170^

Differences between some of the signaling pathways used in blastocysts and blastoids have been identified.^169^ Although blastoids mimic aspects of trophectoderm specification,^9^ they do not fully recapitulate specification in embryos, which warrants further investigation. It is also unclear whether blastoids contain a bona fide ICM (expressing ICM markers including LAMA4^32^ and PDGFRA^23^), and if so, whether the mechanisms of hypoblast specification are shared between embryos and blastoids. Indeed, hypoblast cells are often generated at low efficiency, and their localization is often inaccurate in blastoids. To what extent blastoids and blastocysts share or diverge with respect to regulatory mechanisms will be the focus of the next decade of research.

Another limitation of blastoids and stem cell embryo models, in general, is that current protocols are not fully optimized and can give rise to cell types that should not be present in the model. These so-called off-target cells include cells that are asynchronous with the normal developmental program, such as amnion-like and ExM-like cells present in some blastoid models.^99,172^ In addition, the developmental potential of mouse and cynomolgus monkey blastoids is currently very limited.^13,191^ In 2021, the International Society for Stem Cell Research (ISSCR) published new guidelines for stem cell research. The transfer of human stem cell-based embryo models to the uterus of either a human or animal host was categorized as prohibited research activity because it is considered unethical.^66,192,193^ Even though the gold standard experiment to test functionality cannot be performed, it is clear that blastoids are not embryos since they do not currently model earlier (morula) or later developmental stages well (see the post-implantation models section).^171^ Therefore, blastoids complement and stimulate, rather than replace, human embryo research.

### Limitations of post-implantation models

At present, most embryo models demonstrate high intra- and inter-experimental variability. For example, the cell type proportions can vary significantly between structures formed within an experiment and deviate from the proportions found in the natural embryo (Table 1). The outcome greatly depends on the model, protocol, starting cell type, and culture media used. Naive PSCs can acquire genomic abnormalities,^194^ an additional confounding effect. Moreover, in models based on the combination of multiple stem cell types, aggregating a defined number of cells remains challenging. Lastly, different groups use slightly different starting media conditions that are all categorized under the umbrella of either naive, intermediate, or primed pluripotency. How these different variables influence the outcome remains poorly understood. Cross-lab sharing of protocols and cells could be an important way for the field to collectively define these features.

Another limitation is the scarcity of reference datasets due to challenges associated with working with human embryos, especially at the post-implantation stage. Improving our knowledge of the embryo, for instance, by providing reference maps at the cellular and molecular level, will be fundamental to enable comparisons with embryo models. These maps will help to determine the advantages and limitations of each model^172^ and which specific questions they can address. An inability to correctly mimic the sequence of cell fate specification, tempo of development, or the final proportion of cell types and state composition could limit the ability of certain models to study aspects of development. Similarly, some models are inefficient, and only a very small fraction of the structures obtained mimic the embryo, whereas the rest substantially departs from a natural morphology. The difference in developmental timing between *in vitro* models and *in vivo* embryos is yet another limitation, as well as the potential asynchrony among different embryo lineages within embryo models.

Finally, the absence of well-defined ethical standards in most jurisdictions opens many questions. For the field to flourish, it is crucial to establish tailored governance frameworks, created specifically to regulate research using human embryo models. These frameworks will be essential to ensure confidence among researchers, ethicists, lawmakers, and the general public that the research is conducted ethically and responsibly.

## Future Outlook

With an increasingly complex toolbox of stem cell-based embryo models and associated strengths and limitations, it will be fundamental to determine which models are relevant for which developmental events. Ultimately, the choice of the model will depend on the specific question that needs to be addressed.

Studying early human embryo development has major implications for human reproduction. It will allow us to improve embryo culture conditions, identify biomarkers for embryo selection, and hence improve ART. Congenital malformations are one of the main causes of childhood mortality, and some of them have their origins at early developmental stages.^195^ In addition, it remains unclear how aneuploidy, the presence of an abnormal number of chromosomes, impacts human embryo development. Aneuploid cells are detected in approximately 80% of *in vitro* fertilized human embryos,^31,107,196^ and embryo models offer a unique opportunity to study their influence during development. This knowledge will be of relevance to our understanding of embryo plasticity and the mechanisms of pregnancy loss.

Advances in embryo culture, computational, and omics techniques will generate atlases of embryos at different developmental stages and facilitate comparisons between models and embryos, as well as model improvements.^172^ Stem cell-based embryo models offer exciting opportunities to tackle important questions. For instance, exploring the contribution of cues from the physical environment during human development is an important future goal. Models that closely mimic the spatial arrangement of tissues present in the human embryo will be ideally suited to study mechanobiological questions.

Human embryo models also lend themselves to the investigation of dynamic events. They are uniquely placed to study the cell types that arise during early human post-implantation, such as PGCs and ExM cells. Addressing this question requires lineage-tracing technology, which is challenging to perform in human embryos. The contribution of the uterine environment to embryo development is another important open question. Complex co-culture systems containing human embryos (or embryo models) and endometrial organoids may provide additional insights.

Clearly, stem cell-based embryo models are not embryos. The cell type composition, proportion, and spatial cellular organization are not perfectly recapitulated. However, the fact that they are imperfect, and hence not embryos, makes them an attractive experimental system, full of advantages and possibilities. Advances in stem cell-based human embryo models not only open an era of significant scientific discovery but also promise to shed light on the causes of pregnancy disorders, making this an extraordinarily exciting time for the field.

## Figures and Tables

**Table 1:** Comparison of published stem cell-based models of the post-implantation human embryo

	Zheng et al.^176^	Weatherbee et al.^10^	Pedroza et al.^11^	Ai et al.^111^	Oldak et al.^12^	Hislop et al.^188^	Liu et al.^181^	Okubo et al.^71^
Starting cell types	primed human PSCs	intermediate human PSCs that are differentiated to epiblast-like, hypoblast-like, and trophoblast-like cells via transgene overexpression	intermediate human PSCs	naive human PSCs and extra-embryonic-like stem cells	naive human PSCs that are differentiated to hypoblast-like, ExM-like, and trophoblast-like cells and combined with naive human PSCs	primed human PSCs combined with yolk sac-like cells generated via transgene overexpression	extended potential human PSCs	naive human PSCs combined with hypoblast-like generated via transgene overexpression
Forced expression of transgene	no	yes	no	no	no	yes	no	yes
Experimental approach	growth in a 3D gel of defined physical properties coupled to a microfluidics device	aggregation and self-organization in suspension	aggregation and self-organization in suspension	aggregation and self-assembly in suspension	aggregation and self-assembly in suspension	self-assembly on 2D culture plates	aggregation and self-organization in suspension	aggregation and self-assembly in suspension
Stage modeled	early gastrulation	early post-implantation	early post-implantation	early post-implantation	early post-implantation	post-gastrulation development of extra-embryonic tissues	gastrulation	early post-implantation
Advantages	robust and reproducible	modularity	easy to set up	modularity and methodology informed by embryonic signaling	modularity and high morphological resemblance to day 12–14 post-implantation embryos	modular and easy to set up	advanced developmental stages reached and easy to set up	modular and easy to set up
**Limitations**								
Implementation	requires microfluidics expertise	differences in the expression levels of the exogenous transcription factors may lead to differences in outcome	N/A	involves several cell types and differentiation protocols	involves several cell types and differentiation protocols	differences in the expression levels of the exogenous transcription factor may lead to differences in outcome	N/A	N/A
Embryonic tissue	N/A	excessive differentiation leading to loss of pluripotent cells and excessive ExM cells	limited post-implantation morphogenesis	N/A	N/A	the epiblast-like domain is developing on 2D plastic	derived from expanded potential stem cells, which have an unclear pluripotent state	N/A
Extra-embryonic tissues	not present	lack of bona fide trophoblast and visceral endoderm cells do not form a yolk sac	no ExM, no trophoblast, and the visceral endoderm does not form a yolk sac	extra-embryonic cell types without an *in vivo* counterpart	incomplete development of the trophoblast	no trophoblast	no trophoblast	trophoblast cells are not in direct physical contact with the epiblast, and visceral endoderm cells do not form a yolk sac
Efficiency	high	intermediate	intermediate	intermediate	low	high	intermediate	intermediate
**Suitability for studies**								
Study of cell fate decisions and tissue crosstalk	yes	yes	yes	yes	yes	yes	yes	yes
Study of tissue morphogenesis	limited to epiblast and amnion	limited to epiblast and amnion	limited to epiblast and amnion	limited to epiblast, amnion, and yolk sac	limited to epiblast, amnion, and yolk sac	limited to epiblast, amnion, and yolk sac	limited to epiblast, amnion, and yolk sac	limited to epiblast and amnion
High-throughput studies	yes	no	no	no	no	yes	no	no
Labeling individual starting cell types to dissect tissue of origin	no	yes	no	yes	yes	yes	no	yes

## Box 1 Definitions

**Amnion:** a thin extra-embryonic membrane that forms from the epiblast shortly after implantation and gives rise to a fluid-filled amniotic cavity that surrounds the developing embryo.

**Blastocyst:** stage of pre-implantation mammalian embryogenesis in which an outer layer of trophectoderm cells forms a cavity containing an inner cell mass. The latter then forms the epiblast and hypoblast.

**Blastoid:** a stem cell model of the blastocyst.

**Cell fate specification:** a reversible process that restricts the potential of a cell toward a particular cellular identity, such that cells can differentiate toward alternative fates when in the appropriate environment. A cell is specified if it can give rise to its defined lineages when isolated in culture.

**Cell fate commitment:** an irreversible process that restricts the potential of a cell to a particular cellular identity, such that cells can no longer switch to another lineage even when exposed to different cues or placed into a different environment.

**Compaction:** the first morphogenetic event that takes place during embryo development and leads to a tighter conformation of the embryo.

**Conceptus:** the fetus and its extra-embryonic membranes.

**Cytotrophoblast:** inner layer of trophoblast cells that develops upon implantation of the embryo into the uterus and contains the stem cells of the placenta.

**Epiblast:** the embryonic tissue that becomes specified prior to implantation and forms nearly all fetal cell lineages and some extra-embryonic membranes, such as the amnion.

**Extra-embryonic mesoderm (ExM):** mesenchymal cells that start to appear in the human embryo before gastrulation and go on to line the inner surface of the cytotrophoblast, yolk sac, and amnion, form a stalk that connects the embryo and cytotrophoblast (primitive umbilical cord), and later form the primary blood.

**Extended pluripotency:** a state of pluripotency in *in vitro* cultured cells that captures molecular and functional features of morula-stage embryos. Some of the extended pluripotency cultures reported thus far have been later shown to display a peri-implantation pluripotent character.

**Formative pluripotency:** an intermediate state between naive and primed pluripotency. It is defined as a state of competency for germ layer differentiation and represents the early post-implantation epiblast.

**Hypoblast:** a layer of cells below the pre-implantation epiblast in the blastocyst embryo. Hypoblast cells go on to form the yolk sac in post-implantation stages.

**Inner cell mass (ICM):** the group of cells contained within the outer layer of cells that forms the blastocyst. Shortly before implantation, it gives rise to epiblast and hypoblast.

**Morula:** a developmental stage that precedes the blastocyst and is characterized by the presence of a group of blastomeres that result from cleavage of the zygote.

**Naive pluripotency:** a state of pre-implantation pluripotency devoid of lineage biases. In humans, naive pluripotent cells can form all embryonic and extra-embryonic derivatives *in vitro*.

**Pluripotency:** the capacity to give rise to all differentiated cells of the body and germ line.

**Primitive endoderm:** see hypoblast.

**Polar trophectoderm:** trophectoderm cells of the blastocyst located in close proximity to the ICM.

**Mural trophectoderm:** trophectoderm cells of the blastocyst located farthest from the ICM.

**Primed pluripotency:** a state of post-implantation pluripotency characterized by the presence of lineage biases.

**Self-assembly:** the process by which an initial heterogeneous group of cells arranges into an organized structure or pattern.

**Self-organization:** the process by which an initial homogenous group of cells generates diverse cell types that arrange themselves into an organized structure or pattern.

**Stem cell-based embryo model:** an integrated group of different cell types that recapitulates transcriptional, cellular, and morphological features of the embryo.

**Totipotency:** the ability of a single cell to give rise to all differentiated cell types of the conceptus (see Box 2).

**Trophectoderm:** outer epithelial layer of the blastocyst that forms the trophoblast upon embryo implantation.

**Trophoblast:** all cells derived from the trophectoderm.^17^ This term was coined in 1889 to denote cells that transport nutrients from the maternal blood to the fetus.^18^

**Yolk sac:** post-implantation extra-embryonic membrane that forms a sac outside of the embryo. It has several key functions during embryonic development, such as providing nutrition and gas exchange between the mother and the developing embryo, before the placenta forms. The yolk sac is the site of primary blood formation and regresses at later stages of development.

## Box 2 Experimental criteria to evaluate human totipotency

Defining totipotency relies on criteria of variable stringency.^64^ Below, we describe criteria of increasing stringency that can be used to verify totipotency in humans when evaluating candidate human totipotent stem cells.

**Transcriptional resemblance to pre-implantation embryos:** gene expression analyses can be used to assess cellular states and marker gene expression. One criterion is to confirm whether genes expressed in early human pre-implantation blastomeres are also expressed in candidate totipotent stem cells. Genes of blastomeres must be expressed. Analyses of key protein markers can also be used. Another more stringent test is to compare the entire transcriptome using correlation analyses and by aligning single-cell transcriptomes to human embryo reference atlases.

***In vitro* differentiation potential to give rise to all founding lineages:** a test to assess totipotency functionally is to evaluate the capacity of cells to create the three founding lineages, trophectoderm, epiblast, and hypoblast. This ability can be assessed *in vitro* in 2D by switching cells to different culture conditions. This analysis should include controls, such as naive and primed human PSCs, to enable assessing the efficiency of cell fate induction. Efficiency can be assessed by different assays, including real-time quantitative PCR, immunofluorescence, flow cytometry, or single-cell RNA sequencing.

***In vitro* potential to form blastoids:** another, yet more stringent totipotency test is the ability to form blastoids by self-organization. Blastoids should have the appropriate size and morphology and contain cells of the three founding lineages: trophectoderm, epiblast, and hypoblast. By this criteria, human naive PSCs are totipotent. A highly stringent criterion is the formation of blastoids from a single candidate human totipotent stem cell.

**Interspecies chimaera contribution potential:** the developmental potential of candidate human PSCs may be tested by interspecies chimeric assays. The cells are combined with animal embryo cells, and the resulting embryo is analyzed at later stages to evaluate the contribution of candidate totipotent stem cells to the resulting embryo. Totipotent stem cells should not only contribute to distinct lineages, including embryonic and extra-embryonic lineages, but should also express correct marker genes of each lineage. In some cases, it may be necessary to inactivate certain genes, such as p53, which decreases the stringency of the assay. Interspecies differences could limit the usefulness of this assay.

***In vivo* potential:** the most stringent test for totipotency in humans would be the ability of a cell to form a viable baby. This was tested by chance when a pregnancy was achieved from a four-cell stage human embryo with only one surviving blastomere, giving rise to a healthy baby and demonstrating the totipotent state of a four-cell stage human blastomere.^65^ However, the gestation of human stem cell-based embryo models is strictly prohibited in the guidelines published by the International Society for Stem Cell Research (ISSCR), and there is broad international consensus that such experiments would be unethical.^66,67^ Therefore, it is not currently possible to test, by the most stringent criteria, if a candidate human cell is truly totipotent.
